# Counting the Cost: Examining Out-of-Pocket Spending on Emergency Care in Newfoundland and Labrador, Canada

**DOI:** 10.1016/j.acepjo.2026.100451

**Published:** 2026-06-27

**Authors:** Noah Williams, Aswathy Geetha Manukumar, Hensley H. Mariathas, Hai Van Nguyen, Christopher Patey, Shabnam Asghari

**Affiliations:** 1 Centre for Rural Health Studies, Memorial University of Newfoundland, St. John’s, Newfoundland and Labrador A1B 3V6, Canada; 2 Faculty of Pharmacy, Memorial University of Newfoundland, St. John’s, Newfoundland and Labrador A1B 3V6, Canada; 3 Discipline of Family Medicine, Memorial University of Newfoundland, St. John’s, Newfoundland and Labrador A1B 3V6 Canada; 4 Newfoundland and Labrador Health Services, Carbonear Institute for Rural Research and Innovation by the Sea, Carbonear General Hospital, Carbonear, Newfoundland A1Y 1A4, Canada

**Keywords:** emergency medicine, health economics, health equity, out-of-pocket cost

## Abstract

**Objectives:**

To examine patient-reported non–disease-specific out-of-pocket costs (OOPCs) among emergency department (ED) visitors in a publicly funded health care system, including factors associated with OOPC and to explore patient experiences related to these costs.

**Methods:**

This convergent parallel mixed-method study used data collected through telephone surveys and semistructured interviews from March 1, 2021, to July 27, 2023. All patients who visited the 4 EDs (2 rural and 2 urban EDs) in Newfoundland and Labrador, Canada, were randomly selected to participate based on when they visited the ED (date and time). OOPC was defined as the total amount patients incurred from food, transportation, missed work hours, and other related expenses. Quantitative analyses included multivariable binomial and multinomial regression models adjusting for age, gender, ED location, and patient self-reported length of stay (LOS). Sensitivity analyses using multiple imputation assessed the impact of missing data. Qualitative data were analyzed using thematic analysis.

**Results:**

Among the final sample of 818 patients, 24% (199/818) reported OOPC for an ED visit. An ED length of stay of 4+ hours was associated with higher odds of incurring OOPC (adjusted odds ratio [aOR], 2.26; 95% CI, 1.29-4.15) and OOPC of ≥$200 Canadian dollars (CAD; aOR, 3.68; 95% CI, 1.21-11.19). Patients who visited urban EDs were more likely to report OOPC of ≥$200 CAD (aOR, 2.55; 95% CI, 1.09-5.99). Qualitative analysis showed 4 OOPC themes: (1) ED visits are expensive; (2) missing work hours; (3) being forced to choose between care and necessities; and (4) ongoing costs of care.

**Conclusion:**

These findings demonstrate that OOPC persist even within a publicly funded health system and underscore the importance of improving ED efficiency to reduce the financial burden on individuals seeking care.

**The Bottom Line**

Even with a publicly funded health system in Canada, about 1 in 4 patients visiting emergency departments in Newfoundland and Labrador have out-of-pocket costs. Patients who visit urban emergency departments or stay longer than 4 hours are nearly 3 times more likely to pay $200 or more. These hidden costs can force people to choose between paying for basic needs and getting care. Understanding who pays, where, and how much can help hospitals reduce these costs and make emergency care fairer and more accessible for everyone.

## 1 Introduction

### 1.1 Background and Importance

Canada’s health system is publicly financed and provides universal coverage for its approximately 34 million residents, with funding and administration managed by the provinces and territories.^1^^,^^2^ Hospitals are mostly nonprofit and publicly funded, although a small number are privately operated.^1^^,^^2^ Hospitals account for approximately 30% of Canada’s total health expenditure, with the largest share spent on employee compensation.^1^^,^^2^ Medically necessary services, including hospital care, physician visits, diagnostic tests, and medications used during hospitalization, are covered by public insurance.^1^^,^^2^ Other services, such as outpatient prescription drugs, home care, and long-term institutional care, are financed through a mix of public programs, private insurance, and out-of-pocket payments.^1^^,^^2^ Coverage for these services varies across provinces, with some offering universal access for seniors and others using income-tested eligibility.^1^^,^^2^ Routine dental care, physiotherapy, and vision care are largely privately financed.^1^^,^^2^ While many Canadians have supplemental insurance to cover some of these costs, a significant portion of the population, particularly those with lower incomes, must pay out of pocket.^1^^,^^2^ Spending on these services has been steadily increasing, highlighting gaps in financial protection and equity.^1^^,^^2^

An increase in staffing difficulties, a growing number of visitors each year and the subsequent increase in patients’ length of stay (LOS) have led Canada’s emergency departments (EDs) to be overburdened.^3^ Outside of the negative systemic impacts of ED overcrowding and increased LOS, an inefficient ED can also adversely affect patient outcomes.^3^, ^4^, ^5^ When considering patient outcomes in Canadian ED research, few studies have chosen to collect information on the adverse financial impacts of ED visits in Canada. This can be partially attributed to the coverage of many direct health care costs in a publicly funded health care system.^6^ Extraneous direct costs or indirect costs are costs not covered under the Canadian health care system, defined in this study as out-of-pocket costs (OOPCs). OOPCs are expenses borne directly by patients where neither public nor private insurance covers the full cost of the health good or service.^7^ This study highlights the often-overlooked financial burden on patients and families, emphasizing the need for health care systems to account for and address these OOPCs.

### 1.2 Goals of This Investigation

We explored the OOPC incurred by patients visiting 4 geographically diverse Canadian EDs. OOPC included the proportion, amount, and types of OOPC and covariates influencing OOPC in the ED. Covariates of interest were gender, age, ED location (rural vs urban), and LOS.

## 2 Methods

This study has received ethics approval from the Health Research Ethics Board of Newfoundland and Labrador (HREB No. 2019.264).^4^^,^^5^^,^^8^^,^^9^

### 2.1 Study Design and Setting

This multisite, convergent parallel mixed-method study^10^ included 2 rural and 2 urban EDs with continuous physician coverage.^4^^,^^5^^,^^8^^,^^9^ The rural sites were smaller units serving their surrounding communities, supported by multidisciplinary clinical teams and access to specialist consultation.^5^^,^^11^ Urban sites included larger centers offering comprehensive emergency, inpatient, outpatient, and tertiary-level services and functioned as referral hubs for the broader provincial population.^5^^,^^11^ Only one of the urban EDs operated a fast-track urgent care area; patient assessment at the remaining sites occurred within standard emergency care spaces.

### 2.2 Data Collection and Participant Selection

Data collection began on March 1, 2021, and concluded on July 27, 2023.^4^^,^^5^^,^^8^^,^^9^ We used a survey for data collection.^4^^,^^5^^,^^8^^,^^9^ The telephone survey was developed using established patient experience instruments with demonstrated validity such as the Ontario Emergency Department Patient Experience of Care Survey and the Press Ganey Emergency Department Survey.^4^^,^^5^^,^^8^^,^^9^ Using a semi-Delphi approach with the research team and patient research partners (PRPs), we refined and assessed the questionnaire for content, construct, and face validity.^4^^,^^5^^,^^8^^,^^9^ PRPs are patients or caretakers who have actively engaged in this study since its initial stages.^4^^,^^5^^,^^8^^,^^9^ We also pilot tested the survey with 10 volunteers, and their responses were used for further validation.^4^^,^^5^^,^^8^^,^^9^

Data were originally collected as part of a larger study to improve ED efficiency.^4^^,^^5^^,^^8^^,^^9^ Patient selection was done using a random date/time generator based on the visit.^4^^,^^5^^,^^8^^,^^9^ All patients who visited the ED during the study period, irrespective of their admission or discharge status, were eligible to participate in the study. They were contacted by the research assistant (RA) within 48 hours of leaving the ED.

Participants were contacted by a trained RA to complete the 30-minute telephone survey, and data were entered directly into Qualtrics.^4^^,^^5^^,^^8^^,^^9^^,^^12^ Every month, the RAs reached out to at least 25 participants from each of the 4 sites, with an expectation of at least 50% response rate.^4^^,^^5^^,^^8^^,^^9^ If 1 of the selected participants was unavailable, the RA contacted them at least 3 times before contacting the next patient.^4^^,^^5^^,^^8^^,^^9^ If there was no ED visit on the chosen date and time (eg, April 5, 2022, at 10:50 am), we used a ±2-hour window to select the next patient (eg, a patient who visited at 11:30 am on the same day).^4^^,^^5^^,^^8^^,^^9^

Patients who showed interest in the follow-up interviews were contacted later, and their responses were recorded and verbatim transcribed.^4^^,^^5^^,^^8^^,^^9^ These participants were selected through theoretical sampling.^13^ At the end of the telephone survey, patients were asked whether they consented to being contacted by our research team for a second in-depth interview lasting 45 minutes to discuss further details of their visit.^4^^,^^5^^,^^8^^,^^9^ Patients who agreed were contacted via telephone later and those who completed these semistructured interviews received a gift card of $25 CAD for their participation.^4^^,^^5^^,^^8^^,^^9^

### 2.3 Outcome Variables

OOPC in this study included the OOPC reported for an ED visit, the amount of OOPC for the visit, and the types of OOPC incurred. The OOPC reported was organized as whether or not these costs were reported. The amount of OOPC was collected in levels of $0, $1 - $199, $200 and more and was in Canadian Dollars (CAD). The types of OOPC include missed hours of work, ambulance, taxi, and childcare provider/caretaker.

### 2.4 Covariates

The covariates controlled for this analysis included age, gender, location and patient-reported LOS. Age was categorized into youth (0-20 years), working age (21-65 years), or retired (66+ years). Patients' self-reported gender is organized in this study as a binary variable (males vs females). The hospital location was determined based on those who visited rural vs urban EDs. LOS is defined as the total amount of time for an ED visit and includes <2 hours, 2 to 4 hours, and 4 hours or more. LOS was collected using the question: “Overall, what was the total length of your visit to the ED?”.

### 2.5 Analysis

In this convergent parallel design,^10^ quantitative and qualitative data were collected at similar time frames. Quantitative data were collected via telephone surveys, while qualitative data were collected through open-ended survey questions and semistructured interviews. Each data type was analyzed separately at first: quantitative data were examined using regression models by coauthors with statistical expertise, and qualitative data were coded by 5 team members with expertise in qualitative research using thematic analysis. After the separate analyses, qualitative findings were compared with the results of the quantitative analyses to identify similarities and differences, providing a comprehensive understanding of OOPC during ED visits.

#### 2.5.1 Quantitative analysis

The study population was described using frequencies. OOPC was described based on level, type of cost, and proportion of patients incurring OOPC.

Multivariable binomial and multivariable multinomial regression analyses were conducted to determine associations between OOPC and the covariates (age, gender, ED location, and LOS). The association between different levels and types of OOPC and all covariates were also analyzed. Bivariate analysis was tested using Pearson χ^2^ test. Multivariable analyses were tested using binomial and multinomial regressions. A *P* value of <.05 was considered statistically significant. R Studio 2024.04.2^14^ was used for all analysis.

For variables with missing data, we conducted a sensitivity analysis using multiple imputation with chained equations.^15^, ^16^, ^17^ Twenty imputations were performed following recommended guidelines, and binomial and multinomial regression analyses were repeated to assess the impact of missing data on the results.^15^, ^16^, ^17^ The sensitivity analysis is described in detail in File S1.

#### 2.5.2 Qualitative analysis

We used a convergent thematic analysis design^18^ to extract themes and codes related to OOPC during the ED visit. Both open-ended survey responses and semistructured interviews were integrated to provide a comprehensive understanding of patient experiences.^4^^,^^5^^,^^19^ Five researchers independently conducted open coding, ensuring multiple perspectives were applied to the data.^4^^,^^5^^,^^19^ Conflicts in coding and interpretation were resolved through regular analytic meetings, during which themes were refined and definitions clarified.^4^^,^^5^^,^^19^ Survey and interview findings were compared and crossvalidated to strengthen credibility and confirm consistency across data sources.^4^^,^^5^^,^^19^

Thematic saturation was determined based on repeated patterns and ideas observed across both methods and was further verified through team discussions.^4^^,^^5^^,^^19^ Preliminary results were presented to the broader research team, including qualitative health research experts, clinicians, and PRPs, allowing for iterative feedback and validation.^4^^,^^5^^,^^19^ Themes were finalized after multiple rounds of discussion, ensuring that the final interpretations accurately represented participants’ experiences and perspectives.^4^^,^^5^^,^^19^

## 3 Results

### 3.1 Characteristics of Study Subjects

Overall, we contacted approximately 1575 patients during the study period. Of these, 831 completed the survey, corresponding to a response rate of 52.8%. Of the 831 survey respondents, how we reached the final sample size of 818 was already explained in the original article: after excluding patients who did not respond to questions about OOPC (n = 3) or did not remember if they incurred OOPC (n = 10), our final sample size was 818 (Fig).

**Figure:**
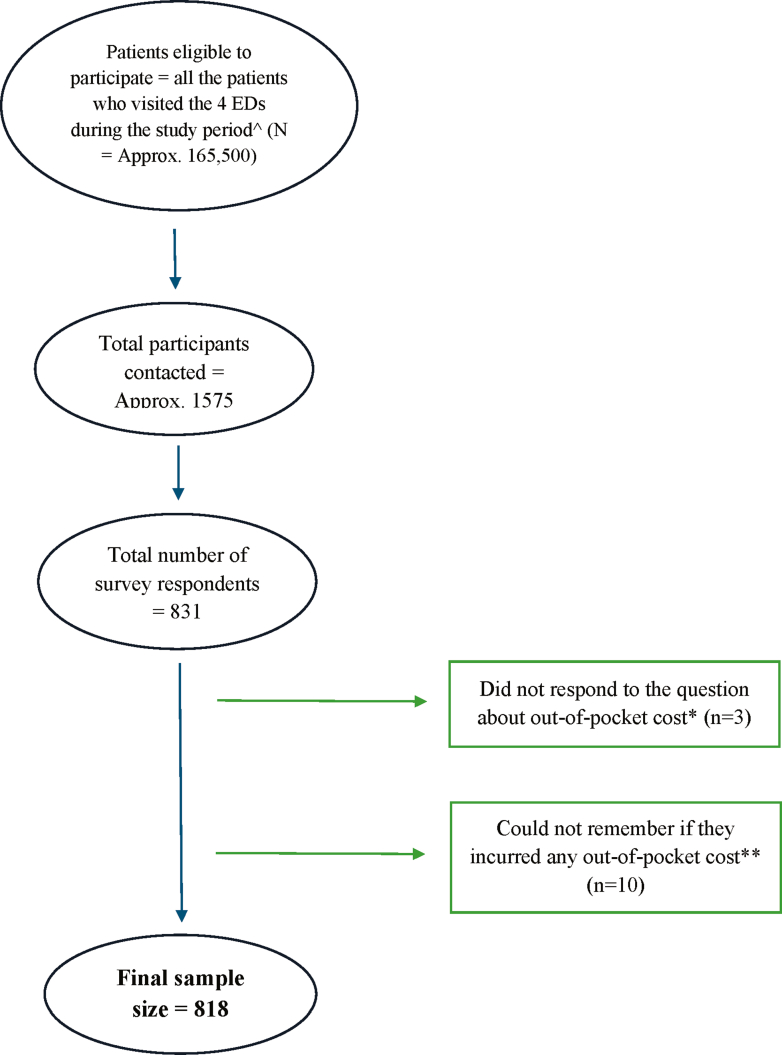
Patient selection flow diagram. ˆStudy period = March 1, 2021, to July 27, 2023. ∗Did not respond to the question: “Overall, how much did the emergency department visit cost you? (This would include food, hotel, transportation, babysitters/caretakers, gas, parking, missed hours of work, etc.).” ∗∗Responded with “I can’t remember” to the aforementioned question.

Most of the patients were aged 21 to 65 years (62%) and were females (62%). Five of these patients also completed the follow-up interviews, at which point we reached data saturation. Of the 5 patients, 3 were females, and 2 were males, aged between 40 and 70 years.

### 3.2 Quantitative Analysis

OOPC was reported by approximately 24% (n = 199/818) of patients for an ED visit (Table 1).

**Table 1:** OOPC breakdown of patients who visited 4 EDs in Newfoundland between March 2021 and July 2023 (N = 818).

OOPC variable	Value, n (%)
OOPC	
No	619 (76)
Yes	199 (24)
OOPC level^a^ ($)	
0	619 (76)
1-199	160 (19.6)
200-499^b^	25 (3)
500-999^b^	5 (0.6)
1000-5000^b^	6 (0.7)
5000+^b^	3 (0.4)
Type of OOPC	
Did not incur OOPC	619 (76)
Missed work hours	51 (6)
Ambulance	37 (4.5)
Taxi^c^	11 (1.3)
Childcare provider/caretaker^c^	5 (0.6)
Unspecified^c^	95 (11.6)

ED, emergency department; OOPC, out-of-pocket cost.

a Cost is in Canadian dollars.

b,c Combined into a single level for further analysis due to sparse data.

#### 3.2.1 Bivariate analysis

Table 2 shows the association between incurring OOPC and all covariates. The χ^2^ test of association showed that OOPC was associated with ED location and LOS (Table 2).

**Table 2:** Factors associated with incurring OOPC for patients who visited 4 EDs in Newfoundland between March 2021 and July 2023 (N = 818).

Covariates	OOPC, n (%)		*P*
	No (*n* = 619, 76%)	Yes (*n* = 199, 24%)	
Age (y)			.10
0-20	49 (8)	9 (5)	
21-65	374 (60)	135 (68)	
66 or older	196 (32)	55 (28)	
Gender			.14
Female	378 (61)	133 (67)	
Male	241 (39)	66 (33)	
ED location			<.001∗
Rural	325 (53)	69 (35)	
Urban	294 (47)	130 (65)	
Total length of stay (h)			<.001∗
0-2	197 (32)	18 (9)	
2-4	179 (29)	15 (7)	
4+	229 (37)	51 (26)	
Missing responses^a^	14 (2)	115 (58)	

ED, emergency department; OOPC, out-of-pocket cost.

∗Significance level, *P* < .001.

a Responses lost due to an error in Qualtrics during the data collection period.

#### 3.2.2 Binomial regression analysis

A binomial logistic regression was performed with covariates age, gender, ED location, and LOS. The outcome was dichotomous: Did you incur OOPC during the ED visit? Yes vs no. The analysis showed that patients who stayed in the ED for more than 4 hours had higher odds of incurring cost than those who stayed for less than 2 hours (adjusted odds ratio [aOR], 2.26; 95% CI, 1.29-4.15; *P* = .006) (Table 3).

**Table 3:** Binomial logistic regression for incurring OOPC for patients who visited 4 EDs in Newfoundland between March 2021 and July 2023 (N = 818).

Covariates	Odds of incurring OOPC, aOR (95% CI)
Age (y; reference = 0-20)	
21-65	0.69 (0.31-1.68)
66 or older	0.51 (0.21-1.31)
Gender (reference = female)	
Male	1.07 (0.61-1.72)
Location (reference = rural)	
Urban	1.38 (0.85-2.28)
Length of stay (h; reference = 0-2)	
2-4	0.85 (0.41-1.75)
4+	2.26 (1.29-4.15)∗

aOR, adjusted odds ratio; ED, emergency department; OOPC, out-of-pocket cost.

∗Significance level, *P* < .05.

#### 3.2.3 Multinomial regression analysis

A multinomial regression was run to determine the association between the OOPC level (dichotomized as $0 vs <$200 and ≥$200) and all covariates. We found that patients who were 66 years or older (aOR, 0.18; 95% CI, 0.03-0.93; *P* = .041) were less likely to incur OOPC of $200 CAD or more, compared with those who did not incur any cost. The model also showed that patients who visited urban EDs (aOR, 2.55; 95% CI, 1.09-5.99; *P* = .032), and patients whose LOS was 4+ hours (aOR, 3.68; 95% CI, 1.21-11.19; *P* = .022) had higher odds of incurring OOPC of $200 CAD or more, compared with those who did not incur any cost (Table 4).

**Table 4:** Multinomial regression for level of cost for patients who incurred OOPC while visiting 4 EDs in Newfoundland between March 2021 and July 2023 (N = 818).

Covariates	Level of OOPC	
	$0 vs <$200, aOR (95% CI)	$0 vs ≥$200, aOR (95% CI)
Age (y; reference = 0-20)		
21-65	0.65 (0.23-1.81)	0.69 (0.19-2.51)
66 or older	0.74 (0.25-2.13)	0.18 (0.03-0.93)∗
Gender (reference = female)		
Male	1.00 (0.55-1.81)	1.22 (0.59-2.56)
Location (reference = rural)		
Urban	0.99 (0.54-1.81)	2.55 (1.09-5.99)∗
Length of stay (h; reference = 0-2)		
2-4	0.63 (0.26-1.55)	1.58 (0.45-5.57)
4+	1.83 (0.93-3.63)	3.68 (1.21-11.19)∗

Cost level is in Canadian dollars.

aOR, adjusted odds ratio; ED, emergency department; OOPC, out-of-pocket cost.

∗Significance level, *P* < .05.

Similarly, a multinomial logistic regression was run to evaluate the association between the type of OOPC (3 levels: none of these apply vs other; none of these apply vs missed hours of work; none of these apply vs ambulance) and all covariates. The model showed that patients who were 66 years or older (aOR, 0.15; 95% CI, 0.04-0.61; *P* =.01) had lower odds of losing income due to missed hours of work, compared with those who did not incur any OOPC during the ED visit. We also found that patients who visited urban EDs (aOR, 2.47; 95% CI, 1.20-5.09; *P* =.01) were more likely to have missed hours of work as the type of OOPC incurred (Table 5).

**Table 5:** Multinomial logistic regression for type of cost for patients who incurred OOPC while visiting 4 EDs in Newfoundland between March 2021 and July 2023 (N = 818).

Covariates	Type of OOPC		
	None of these apply vs other, aOR (95% CI)	None of these apply vs missed hours of work, aOR (95% CI)	None of these apply vs ambulance, aOR (95% CI)
Age (y; reference = 0-20)			
21-65	1.33 (0.16-10.88)	0.59 (0.21-1.65)	0.51 (0.10-2.58)
66 or over	1.40 (0.16-12.11)	0.15 (0.04-0.61)∗	0.96 (0.20-4.69)
Gender (reference = female)			
Male	0.78 (0.29-2.12)	0.81 (0.42-1.59)	2.38 (0.96-5.92)
Location (reference = rural)			
Urban	0.77 (0.29-2.07)	2.47 (1.20-5.09)∗	0.80 (0.32-2.01)
Length of stay (h; reference = 0-2)			
2-4	0.69 (0.16-2.98)	0.97 (0.36-2.62)	0.72 (0.17-3.08)
4+	1.81 (0.59-5.55)	2.27 (0.98-5.26)	2.49 (0.85-7.28)

aOR, adjusted odds ratio; ED, emergency department; OOPC, out-of-pocket cost.

∗Significance level, *P* < .05.

### 3.3 Qualitative Analysis

We analyzed 823 responses for the qualitative analysis, including 818 open-ended survey responses and semistructured interviews with 5 of these patients, achieving thematic saturation while capturing maximum variability. Thematic analysis of the open-ended survey questions and semistructured interviews led to 4 themes around OOPC: (1) ED visits are expensive; (2) missing work hours; (3) being forced to choose between care and necessities; and (4) ongoing costs of care. Additionally, we found that those who visited Urban EDs raised more concerns about OOPC. Our PRPs agreed that these themes reflect their lived experiences.

#### 3.3.1 ED visits are expensive

Patients reported having OOPC due to high medication costs and high ambulance costs.

[Patient] has been through hell in the past year with the medical system … has had [issues] with getting sick leave pay as well as a drug card to cover her prescriptions … social services told her that there was nothing they could do…—survey response from a female patient, 21 to 65 years, who visited a rural ED.

Ambulances should not cost as much as they do…*—*survey response from a male patient, 21 to 65 years, who visited an urban ED.

Conversely, some patients did not report incurring OOPC. For example, patients who lived nearby or had family support could avoid transportation and childcare provider/caretaker costs.

I live about 5 minutes from the hospital [so there was no cost for transportation]—semistructured interview response from a male patient, 21 to 65 years, who visited a rural ED.

I have a daughter here in town … a couple of daughters [so did not pay for a caretaker]—semi-structured interview response from a female patient, 66 years or older, who visited an urban ED.

#### 3.3.2 Missing work hours

Patients reported missing several hours to multiple days of work and shared challenges in accessing sick leave pay, further contributing to OOPC.

Not everybody has the ability (time off, money, childcare provider, etc) to take time off to wait 8 hours … should not have to choose between health and work.—survey response from a female patient, 21 to 65 years, who visited a rural ED.

#### 3.3.3 Having to choose between care and necessities

Several patients reported being forced to choose between care and necessities due to high OOPC and increased LOS.

People on a fixed income should not have to make a choice between getting the health care they deserve or rent.—survey response from a male patient, 21 to 65 years old, who visited an urban ED.

#### 3.3.4 Ongoing cost of care

Patients reported ongoing OOPC for care due to multiple visits and a lack of support from the health care system.

[Patient] talked about being asked to leave the ED and go back later that day to receive tests … her support person needed to take extra time off work that day to go back to the ED with her. [Patient] talked about being on permanent disability and not receiving any rehabilitation support from the health care system to help her return to work, and therefore she has lost significant income.—survey response from a female patient, 21 to 65 years, who visited an urban ED.

This patient reported spending more than $5000 on OOPC.

## 4 Limitations

Our study has several limitations. First, this study was specifically for non–disease-specific OOPC within a health care system that covers most medical-related costs. Therefore, the variables were selected based on prior studies showing these factors to be related to non–disease-specific OOPC.^20^, ^21^, ^22^, ^23^ However, as this study was embedded within a broader investigation of ED patient experiences, the selection of covariates was partially constrained by the objectives and design of the parent study. Although informed by prior literature, some potentially relevant variables may not have been included due to the need to limit survey length and reduce participant burden. As with many studies using self-reported data, the results of this study might have been biased due to nonresponse, recall bias, and social desirability in responses. Although the survey achieved a 52.8% response rate, characteristics of nonrespondents were unavailable, limiting our ability to assess whether respondents were fully representative of the target population. Participation might have been outcome dependent, with patients experiencing poorer care or higher OOPCs more likely to respond. This differential nonresponse could introduce selection bias and may have influenced the observed associations. Due to patient confidentiality, we did not have access to information on nonrespondents or their reasons for nonparticipation; therefore, findings should be interpreted with caution. We hoped to mitigate nonresponse and recall bias by contacting the patients multiple times within 2 weeks of the initial ED visit regarding interest in participation. Patient anonymity with survey responses was maintained to reduce the risk of social desirability bias.

Surveys can also be susceptible to bias based on data collection, including patient selection, interviewer bias, and information bias. Patient selection bias was mitigated using a random date–time generator based on ED admission time to select participants. Employees were trained in administering interviews to reduce interviewer bias.^24^ A potential lack of external validity of the study was also considered, as this study was limited to data from Canada and only 1 of the Canadian provinces. While research findings are not representative of other health systems that are primarily privatized and incur higher expenses, this article serves as a demonstration of how publicly funded health care systems still contain costs to patients that may be creating barriers.

Age was categorized as youth (0-20 years), working age (21-65 years), and retired (≥66 years), and LOS was categorized as 0 to 2 hours, 2 to 4 hours, and ≥4 hours. These categorizations may have resulted in some loss of information and should be considered when interpreting the results. Finally, LOS data were missing for 16% (129/199) of patients, of whom 58% (115/199) incurred OOPC. These missing responses resulted from a Qualtrics malfunction, the software used to store telephone survey responses, and were therefore considered missing completely at random.^15^ To assess the potential impact of this missing data, we conducted a sensitivity analysis using multiple imputation^15^, ^16^, ^17^ and repeated the multivariable analyses. Overall, the key significant associations observed in the original dataset remained after multiple imputation, suggesting that these findings were robust and not driven by missing data (File S1). It is also worth noting that LOS used in this study was self-reported and might have been affected by recall bias.

## 5 Discussion

Approximately 1 in 4 patients (24%; 199/818) who visited EDs in Newfoundland reported incurring OOPCs, with the majority of these expenditures ranging from 1 to 199 CAD. Conversely, most patients (76%; 619/818) did not report any OOPC, highlighting that while OOPC affect a minority of patients, the financial burden for those impacted may still be meaningful. The average annual income of residents of Newfoundland in 2022 was $51,100,^25^ which equates to $25.55 per hour for full-time employees receiving mandatory 2 weeks of annual vacation. With patients reporting OOPC of 200 CAD or more, an ED visit in Newfoundland is at least 1 day of full-time work. Over one-quarter (26%) of Canadians reported an inability to cover an unexpected expense of $500 in 2022,^26^ burdening patients from incurring OOPC for an ED visit.

Patients who stayed in the ED for longer than 4 hours were found to be more likely to incur OOPC. Visiting urban EDs was also related to patients incurring OOPC of $200 CAD or more. Thematic analysis results also support this interpretation. Most of the patients who commented on OOPC had visited urban EDs and had long LOS. Their narratives highlighted concerns about ambulance costs, lost income from extended time in the ED, and financial strain from balancing health care costs with essential living expenses such as rent.

Studies have shown that since an increasing number of high-acuity patients visit urban EDs, it creates a longer LOS in Canada.^27^ In the bivariate analysis using the original dataset, the χ^2^ test showed that OOPC was associated with ED location (χ^2^ = 19.2; degrees of freedom = 1; *P* < .001); however, this association was not observed in the adjusted binomial regression model. In contrast, the binomial regression conducted as part of the sensitivity analysis using multiple imputation (m = 20) showed that patients who visited urban EDs had a higher odds of incurring OOPC than those who visited rural EDs (aOR, 1.77; 95% CI, 1.24-2.52; *P* = .002) (File S1). This finding suggests that missing data may have contributed to underestimating the association between ED location and OOPC in the complete-case analysis after adjustment for age, gender, and LOS. Furthermore, the fact that associations observed in bivariate analyses were not retained in multivariable models may reflect confounding and shared variance among predictors, thereby reducing the independent effect of a variable after adjustment for related covariates.^28^ In addition, collinearity between variables may further attenuate these associations.^28^

Of the 818 patients who participated in this study, 199 reported incurring OOPC. This rate was based on the indirect OOPC reported for an ED visit (excluding OOPC such as insurance copayments, deductibles, or hospital expenses). Only 1 study discussed all-cause indirect OOPC, excluding insurance copayments, deductibles, or hospital expenses, and found that 26% of patients incurred OOPC.^29^ This was comparable with the findings of our study, which identified that 24% of patients incurred OOPC.

Patients also reported losing significant income due to multiple ED visits, including up to a month of working days. Multiple studies reported similar levels of cost missed,^30^^,^^31^ while 2 studies reported much lower OOPC due to productivity loss.^32^^,^^33^ Our results showed that patients who were aged 66 years or older were less likely to lose income during their ED visit, while those who visited urban EDs were more likely to miss work and incur OOPC.

There was no significant association between LOS and levels of OOPC. Mahony et al^20^ identified a connection between LOS and OOPC, where decreased LOS in the ED also resulted in decreased OOPC. While longer ED stays were significantly associated with higher odds of incurring OOPC in this study, the practical effects on individual patients may differ. These findings point to opportunities for system-level improvements; however, further research is needed to evaluate the clinical relevance and economic burden at the patient level.

The rate of patients incurring transportation costs varies across the literature. Sørensen et al^34^ reported 43% of patients incurring transportation OOPC, while Giaquinto et al^35^ reported between 45% and 98% of patients incurring transportation OOPC across 5 countries. Qualitative analysis showed that most transportation costs were incurred due to high ambulance rates and paid parking.

This study highlights the importance of implementing strategies to mitigate OOPC in the ED. Literature has suggested that a slight increase in local taxes could help reduce the level of OOPC for an ED visit when health systems are publicly funded.^36^ A community hospital transportation system was also recommended to reduce the level of OOPC for transportation to the ED.^33^^,^^37^ The use of community paramedic services, modifications of nonemergent medical transportation, and innovative ridesharing options were recommended in the literature to help reduce higher ambulance costs for an ED visit from a rural area.^38^ A medical financial assistance program, such as the Medical Transportation Assistance Program, for specialized medical procedures,^39^ is another solution that could be implemented for those who do not have access to adequate emergency services in their area. Improving patient LOS can also help lower costs.^20^ A study using an alternate ED observation pathway based on patients’ acuity showed a reduction in the average LOS for ED patients and a subsequent lowering of OOPC.^20^

As a novel study in Newfoundland, this research starts a discussion highlighting that OOPCs are being incurred, even in a “free” Canadian health system. Approximately 1 in 4 patients incur OOPC for non-disease-specific ED visits from multiple sources, and patients who stay in the ED for 4+ hours are more likely to incur OOPC for an ED visit. Now is the time for new and innovative solutions that target LOS in the ED, creating an efficient environment where patients do not need to take a lot of time off work or wait multiple hours to receive treatment. In this economy, the loss of even a single day’s income can represent a significant financial burden for individuals and families. Strategies such as implementing cost assistance programs, alternate observation pathways, and ED management systems could benefit patients by lowering the costs of staying in the ED. Future initiatives should consider integrating routine monitoring of patient financial burdens into ED quality metrics and implement strategies to mitigate these expenses, improving transparency, and a more comprehensive, patient-centered approach to care delivery.

## Author Contributions

SA, NW, and AGM contributed to the study design, data collection, analysis, and drafting of the manuscript. All authors contributed to data interpretation, critical review and manuscript editing.

## Funding and Support

The SurgeCon project is funded by the 10.13039/501100000024Canadian Institutes of Health Research (grant SR4-165123), the Government of Newfoundland and Labrador (grant 5404-2312-101), Newfoundland and Labrador Health Services, Memorial University of Newfoundland, and the Trinity Conception Placentia Health Foundation.

## Conflict of Interest

CP is one of the cofounders of SurgeCon Innovations. Our team received funding for a project entitled “SurgeCon: An Emergency Department Surge Management Platform” from the Canadian Institutes of Health Research, Newfoundland and Labrador Provincial Government, Newfoundland and Labrador Health Services, and the Trinity Conception Placentia Health Foundation. The other cofounder has a royalty-sharing agreement with MOBIA Technology Innovations and the Newfoundland and Labrador Health Services.
