# Supplementary material for: Counting the Cost: Examining Out-of-Pocket Spending on Emergency Care in Newfoundland and Labrador, Canada
Source: J Am Coll Emerg Physicians Open. 2026 Jun 27;7(4):100451. doi: 10.1016/j.acepjo.2026.100451 (PMC13329481; doi:10.1016/j.acepjo.2026.100451)
Supplement: Supplementary Material [file mmc1.docx]

**Sensitivity Analysis – Multiple Imputation**

**Methodology**

For the sensitivity analysis, we used multiple imputation for the variable Length of Stay (LOS), which had 15.8% (129/818) missing responses.^1^ This data was lost due to a malfunction in the survey software during the initial stages of data collection; therefore, we designated the missing data as MCAR (missing completely at random).^2^

After assessing the missing data, we performed multiple imputation on the LOS data. We used the recommendations of White, Royston, and Wood,^3^ to determine the number of imputations, i.e., the number of imputations should be at least the percentage of incomplete cases. We used m = 20 imputation iterations to achieve convergence.^4^ The R package Multivariate Imputation by Chained Equations (MICE) was used to perform multiple imputation in R Studio version 4.4.1.^5^

Once multiple imputation was completed, we repeated the multivariable binomial and multinomial regressions to assess the impact of missing data on the statistical significance of the findings.

**Results**

The results of the multivariable binomial and multinomial regressions using the data after multiple imputation for LOS are shown in Tables 1, 2, and 3.

The binomial regression for odds of incurring OOPC (no vs. yes) showed that patients who visited the urban EDs were more likely to incur OOPC than those who visited rural EDs (aOR: 1.77, 95% CI: 1.24–2.52, p=0.002); Table 1. Similarly, patients who reported total LOS of ≥4 hours were twice more likely to incur OOPC (aOR: 2.14, 95% CI: 1.29–3.57, p=0.004) than patients with LOS of ≤2 hours.

**Table 1.** Multiple Imputation Multivariable Binomial Regression for Incurring OOPC for Patients who Visited Four EDs in Newfoundland and Labrador, Canada between March 2021 and July 2023 (n = 818)

| **Covariates** | **Odds of incurring OOPC** |
| --- | --- |
|  | **aOR (95% CI)** |
| **Age** (ref*. =* 0-20 years) |  |
| 21 – 65 | 1.38 (0.64–2.97) |
| 66 or over | 1.24 (0.56–2.75) |
| **Gender** (ref*. =* Female) |  |
| Male | 0.79 (0.56–1.12) |
| **ED location** (ref.*=* Rural) |  |
| Urban | 1.77 (1.24–2.52)* |
| **Length of Stay** (ref.*=* <2 hours) |  |
| 2–4 hours | 0.86 (0.44–1.68) |
| 4+ hours | 2.14 (1.29–3.57)* |

OOPC = out-of-pocket cost; aOR = adjusted Odds Ratio; CI = confidence interval; ref*.* = Reference

*Significance at p<0.05

The multinomial regression to examine the association between levels of OOPC (CAD $0 vs <$200 and $0 vs ≥$200 showed significant results for age, ED location and LOS. Older age (66 years or over) was associated with lower odds of incurring OOPC of ≥$200 CAD (aOR: 0.17, 95% CI: 0.04–0.74, p=0.02) compared to younger patients (0-20 years old); Table 2. However, visiting urban EDs was associated with higher odds of incurring both OOPC of ≤$200 CAD (aOR: 1.64, 95% CI: 1.12–2.41, p=0.01) and ≥$200 CAD (aOR: 2.51, 95% CI: 1.15–5.48, p=0.02) compared to patients who visited rural EDs. Finally, patients who reported LOS of 4+ hours were at least twice more likely to incur OOPC of ≤$200 CAD (aOR: 1.96, 95% CI: 1.12–3.42, p=0.02) and ≥$200 CAD (aOR 3.62, 95% CI 1.21–10.84, p=0.02) compared to those who reported a LOS of 0-2 hours.

**Table 2.** Multiple Imputation Multivariable Multinomial Regression for Level of Cost for Patients who Incurred OOPC While Visiting Four EDs in Newfoundland and Labrador, Canada, between March 2021 and July 2023 (n = 818)

|  | **Level of OOPC** | |
| --- | --- | --- |
| **Covariate** | **$0 vs <$200** | **$0 vs ≥$200** |
|  | **aOR (95% CI)** | **aOR (95% CI)** |
| **Age** (ref. = 0-20 years) |  |  |
| 21 – 65 | 1.98 (0.75–5.21) | 0.59 (0.19–1.87) |
| 66 or over | 2.12 (0.79–5.70) | 0.17 (0.04–0.74)* |
| **Gender** (ref. = Female) |  |  |
| Male | 0.71 (0.48–1.04) | 1.26 (0.64–2.49) |
| **Location** (ref. = Rural) |  |  |
| Urban | 1.64 (1.12–2.41)* | 2.51 (1.15–5.48)* |
| **Length of Stay** (ref. = 0-2 hours) | | |
| 2-4 hours | 0.76 (0.36–1.60) | 1.63 (0.48–5.55) |
| 4+ hours | 1.96 (1.12–3.42)* | 3.62 (1.21–10.84)* |

OOPC = out-of-pocket cost; aOR = adjusted Odds Ratio; CI = confidence interval; $ = Canadian Dollars; ref*.* = Reference

*Significance at p<0.05

Finally, we ran a multivariable multinomial regression to evaluate the association between OOPC type and all covariates, using multiple imputation data (Table 3). Older patients (≥66 years) were more likely to report other types of expenses (aOR: 4.51, 95% CI: 1.04–19.55, p=0.04) and less likely to report missed work hours (aOR: 0.16, 95% CI: 0.04–0.55, p=0.004) as the type of OOPC incurred, compared to their younger counterparts (0-20 years old). Visiting urban EDs was associated with increased likelihood of reporting both other types of OOPC (aOR: 1.75, 95% CI: 1.15–2.65, p=0.009) and missed work hours (aOR: 2.47, 95% CI: 1.25–4.87, p=0.009) compared to those who visited rural EDs. Similarly, patients with longer LOS (4+ hours) were twice more likely to report other types of expenses (aOR: 2.04, 95% CI: 1.08–3.87, p=0.03) and missed hours of work (aOR: 2.36, 95% CI: 1.02–5.49, p=0.045) compared to those who reported LOS of ≤2 hours.

**Table 3.** Multiple Imputation Multivariable Multinomial Regression for Type of Cost for Patients who Incurred OOPC While Visiting Four EDs in Newfoundland and Labrador, Canada, between March 2021 and July 2023 (n = 818)

|  | **Type of OOPC** | | |
| --- | --- | --- | --- |
|  | **None of these apply vs. Other** | **None of these apply vs. Missed hours of work** | **None of these apply vs. Ambulance** |
| Covariates | **aOR (95% CI)** | **aOR (95% CI)** | **aOR (95% CI)** |
| **Age** (ref. = 0-20 years) | | | |
| 21 – 65 | 4.13 (0.97–17.65) | 0.54 (0.21–1.42) | 0.75 (0.08–6.71) |
| 66 or over | 4.51 (1.04–19.55)* | 0.16 (0.04–0.55)* | 1.24 (0.14–11.00) |
| **Gender** (ref. = Female) | | | |
| Male | 0.73 (0.48–1.09) | 0.80 (0.43–1.50) | 1.75 (0.57–5.36) |
| **Location** (ref. = Rural) | | |  |
| Urban | 1.75 (1.15–2.65)* | 2.47 (1.25–4.87)* | 0.65 (0.20–2.13) |
| **Length of Stay** (ref. = 0-2 hours) | | | |
| 2-4 hours | 0.84 (0.37–1.89) | 1.02 (0.38–2.75) | 0.46 (0.05–4.54) |
| 4+ hours | 2.04 (1.08–3.87)* | 2.36 (1.02–5.49)* | 2.43 (0.57–10.31) |

OOPC = out-of-pocket cost; aOR = adjusted Odds Ratio; CI = confidence interval; ref*.* = Reference

*Significance at p<0.05

**Discussion – Comparison with analysis results from the original data set**

In the binomial regression analysis using the original dataset, prolonged ED LOS (≥4 hours) was the only variable significantly associated with the odds of incurring any OOPC. When multiple imputation was applied, the association with LOS of 4+ hours remained statistically significant. In addition, ED location emerged as a significant factor, with patients visiting urban EDs having higher odds of incurring OOPC. This suggests that missing data may have masked the effect of ED location in the original complete-case analysis.

In the first multinomial regression analysis using the original dataset, older age (≥66 years), ED location (visiting urban EDs), and lengthy LOS (≥4 hours) were associated with reporting a higher OOPC ($200 CAD or more) than no OOPC. Specifically, patients aged 66 years or older had lower odds of high OOPC, while those seen in urban EDs and those with LOS of 4 hours or more had higher odds. After multiple imputation, these associations remained, and additional significant associations were identified. Visiting urban EDs and LOS of 4+ hours were also significantly associated with incurring lower OOPC (≤$200 CAD). These findings suggest that missing data may have limited the ability of the complete-case analysis to detect associations with moderate levels of OOPC.

Similar patterns were observed in the second multinomial regression analysis, which examined the odds of incurring different types of OOPC (other costs, missed work hours, and ambulance costs). In the original dataset, older age (≥66 years) and ED location (urban EDs) were significantly associated with reporting missed work hours as the type of OOPC incurred. However, analyses using the imputed datasets identified additional associations. Older age (≥66 years), visiting urban EDs, and prolonged LOS (≥ 4 hours) were all significantly associated with reporting other types of OOPC. In addition, LOS of 4+ hours was associated with higher odds of reporting missed work hours, a finding not detected in the original analysis.

Overall, the results from the multiple imputation analyses were consistent with the significant findings from the original dataset and identified additional associations. This suggests that the key results observed in the complete-case analyses were not driven by missing data, and that multiple imputation helped reveal a broader set of factors, particularly ED location and length of stay, associated with both the likelihood and type of OOPC.

**References**

1. Novotny PJ, Schroeder D, Sloan JA, et al. Do Missing Values Influence Outcomes in a Cross-sectional Mail Survey? *Mayo Clin Proc Innov Qual Outcomes*. 2021;5(1):84-93. doi:10.1016/j.mayocpiqo.2020.09.006

2. Kang H. The prevention and handling of the missing data. *Korean Journal of Anesthesiology*. Published online May 24, 2013. https://www.ncbi.nlm.nih.gov/pmc/articles/PMC3668100/

3. White IR, Royston P, Wood AM. Multiple imputation using chained equations: Issues and guidance for practice. *Statistics in Medicine*. 2010;30;30(4):377–99. doi:10.1002/sim.4067

4. Buuren S van. *Flexible Imputation of Missing Data, Second Edition*. 2nd ed. Chapman and Hall/CRC; 2018. doi:10.1201/9780429492259

5. Posit. Posit. Accessed May 6, 2025. https://www.posit.co/
